# SURFACE METROLOGY A Report on the Fourth International Conference on the Metrology and Properties of Engineering Surfaces, National Bureau of Standards, Gaithersburg, MD, April 13–15, 1988

**DOI:** 10.6028/jres.093.154

**Published:** 1988-10-01

**Authors:** T. V. Vorburger

**Affiliations:** Precision Engineering Division, Center for Manufacturing Engineering, National Bureau of Standards, Gaithersburg, MD 20899

Surface metrology finds application to many branches of engineering ranging from optical surfaces to roadways. This triennial conference, held for the first time outside of England, traditionally attracts delegates with diverse interests from many countries. This year, in addition to the delegates from the United States, there were 20 delegates from England, five from France and others from Canada, West Germany, Austria, Japan, Sweden, and Ireland.

Delegates heard and debated 38 presentations on a wide variety of topics that, in retrospect, one could reorganize into the following general areas: measurement techniques, surface specimens, statistical characterization, production methods, and engineering function. A few of the interesting applications are described below.

One notable application to engineering function was Tabenkin and Parsons’ (paper 38) use of the stylus technique to characterize the dispersion of carbon black agglomerates in rubber, a family of materials that might normally be thought of as too soft for reliable characterization by stylus. Inadequate dispersion leads to reduced product life and other detrimental effects in rubber products. Figure 1 shows the stylus in contact with such a rubber surface. The presence of the agglomerates is evidenced either by discrete peaks in the stylus profiles or by valleys left when the material is separated during the cutting process.

Rao et al. (paper 37) discussed their initial work to construct an expert system for selection of surface texture parameters. It was based on a table of results (table 1) gathered by Bielle and others (private communication) for the French automobile industry. The table relates a set of engineering functions such as static sealing with surface texture parameters that should be specified to optimize the function. However, the set of surface parameters shown in table 1, known as the R&W system, are defined using pattern recognition ideas rather than the conventional time series analysis. This approach to correlating surface function with surface finish is an interesting and controversial one.

The conference also recounted many significant advances in the area of measurement techniques.

The traditional technique for measuring surface texture is the stylus. However, nonstylus techniques are being increasingly studied and used. At this conference, research was presented on the measurement of surface texture by optical focusing, optical interferometry, optical scattering, infrared thermography, ultrasonics, two kinds of capacitance techniques, ellipsometry, and scanning tunneling microscopy.

Some of the most dramatic advances are in the field of interferometry (papers 2, 4, and 10) where height resolution at the Angstrom level can be achieved routinely nowadays.

At the large end of the scale, Pryor (paper 14) introduced a new optical imaging technique, known as D-sight, that can detect flaws and small form deviations with great sensitivity on large objects such as automobile and aircraft production components. Figure 2 shows an example of the sensitivity of the technique for inspection of a car body.

Two kinds of capacitance methods were discussed. The area-averaging approach (fig. 3) was analyzed by Lieberman et al. (paper 11) with a realistic model that took into account both the electrical effects due to each tiny element of the surface and the mechanical flexibility of the sensor itself.

By contrast, Garbini et al. (paper 7) described their fringing field capacitive device for profiling (fig. 4). They showed good agreement of roughness average results measured by their approach with those obtained by the stylus technique.

In the area of statistical characterization, Whitehouse (paper 29) developed an interesting unification of friction measurements and stylus-type surface texture measurements and developed a single formulation of the dynamics of both types of systems. His paper highlighted the difficulties both of measuring surface texture in the presence of friction at the point of contact and of measuring friction of components having surface texture.

Lastly, two new types of surface texture specimens were presented. The specimens discussed by Song (paper 21) (fig. 5) had smooth random surfaces machined in steel with roughness average values ranging from 0.012–0.1 *μ*m. They also had high uniformity from one place to the next in spite of the random surfacing process that had produced them. Berger (paper 20) discussed specimens fabricated by silicon technology. Each wafer surface contained a thermally grown SiO_2_ layer with a single well-controlled thickness but with several feature spacings. The result was a surface consisting of “square waves” (fig. 6) suitable as a step height or pitch standard. Since it had four line spacings, one of them being as small as 6 *μ*m, the sample could also be used to test the quality of stylus tip widths ranging from 1 to 12 *μ*m.

The conference co-chairmen were T. V. Vorburger from NBS and T. R. Thomas from Teesside Polytechnic, Middlesbrough, England. The success of the conference was due in great part to the energy of the organizing secretary, K. J Stout, from the University of Birmingham, England.

## List of Papers Presented at Conference

The proceedings, to be published as several successive issues in the journal, Surface Topography, and as *Proceedings of the Fourth International Conference on the Metrology and Properties of Engineering Surfaces* (Kogan Page, London, 1988), will include most of the presentations referenced below.

### Measurement Techniques

1. Baker, L. R., Areal Measurement of Topography. (SIRA, UK)
2. Biegen, J., and Smythe, R., High Resolution Phase Measuring Laser Interferometric Microscopy for Engine Surface Metrology. (ZYGO, USA)
3. Blessing, G., and Eitzen, D.G., Surface Roughness Sensed by Ultrasound. (NBS, USA)
4. Bristow, T. C., Surface Roughness Measurements over Long Scan Lengths. (Photographic Sciences, USA)
5. Church, E. L., Dainty, J. C., Gale, D. M., and Takacs, P. Z., Measurement of Surface Topography by Interference Microscopy. (U.S. Army, USA/Imperial College, UK)
6. Evans, C. J., and Polvani, R. S., Some Observations of Subsurface Damage in Precision Machined Components. (NBS, USA)
7. Garbini, J. L., Jorgensen, J. E., Downs, R. A., and Kow, S. P., Fringe-Field Capacitative Profilometry. (University of Washington, USA)
8. Gee, A. E., Green, D., and Pain, D., An In-Process Tool Proximity Center for Ultra-fine Machining. (Cranfield, UK)
9. Jordan, D. L., Hollins, R. C., Jakeman, E., and Prewett, A., Visible and Infra-Red Scattering from Well Characterised Surfaces. (RSRE/Univerity College, UK)
10. Lange, S. R., and Bhushan, B., Use of Two- and Three-Dimensional, Noncontact Surface Profiler for Tribology Applications. (WYKO/IBM, USA)
11. Lieberman, A. G., Vorburger, T. V., Giauque, C. H. W., Risko, D. G., and Rathbun, K. R., Comparison of Capacitance and Stylus Measurements of Surface Roughness. (NBS/Extrude Hone, USA)
12. Martin, J., Surface Roughness Measurement by Infrared Thermography. (NBS, USA)
13. Osanna, P. H., and Durakbasa, N. M., Comprehensive Analysis of Workpiece Geometry by Means of the Coordinate Measurement Technique. (Wien, AUSTRIA)
14. Pryor, T. R., Reynolds, R., and Pastorius, W., D-Sight: A Whole-Field Optical Technique for Determination of Surface Form Deviation. (Diffracto, CANADA)
15. Scott, P. J., Developments in Surface Texture Measurement. (Rank Taylor Hobson, UK)
16. Sullivan, P. J., Davis, E.J., and Stout, K. J., The Topographical Characteristics of Micro-Indentations and Their Relevance to Microhardness Testing. (Birmingham, UK)
17. Teague, E. C., Surface Metrology, Emphasize METROLOGY. (NBS, USA)
18. Wayte, R. C., Sayles, R., Tweedale, P. J., and Briscoe, B. J., The Design and Construction of an Inexpensive Laser Optical Profilometer. (Imperial College, UK)
19. Williams, M. W., Ludema, K. C., and Hildreth, D. M., Mueller Matrix Ellipsometry of Practical Surfaces. (University of Michigan, USA)

### Surface Specimens

20. Berger, J., A New Surface Roughness Standard Fabricated Using Silicon Technology. (VLSI, USA)
21. Song, J. F., Random Profile Precision Roughness Calibration Specimens. (CIMM, CHINA)

### Statistical Characterization

22. Kagami, J., Hatazawa, T., Yamada, K., and Kawaguchi, T., Three-Dimensional Observation and Measurement of Worn Surfaces. (Utsunomiya, JAPAN)
23. Radcliffe, S. J., and George, A. F., The Analysis and Presentation of Multi-trace Data and Some Applications in Industrial Research. (CEGB, UK)
24. Roques-Carmes, C., Wehbi, D., Quiniou, J. F., and Tricot, C., Modelizing Engineering Surfaces and Evaluating Their Non-Integer Dimension for Application in Material Science. (LMS-ENSMM, FRANCE)
25. Schneider, U., Steckroth, A., Rau, N., and Hubner, G., An Approach to the Evaluation of Surface Profiles by Separating Them into Functionally Different Parts. (Daimler-Benz, WEST GERMANY)
26. Sherrington, I., and Smith, E. H., Fourier Models of the Surface Topography of Engineering Components. (Preston, UK)
27. Thomas, T. R., and Thomas, A. P., Fractals and Engineering Surface Roughness. (Teesside, UK)
28. Watson, W., and Woods, A., The Three Dimensional Representation of Engineering Surfaces. (Leicester, UK)
29. Whitehouse, D. J., Friction and Surface Measurement. (Warwick, UK)

### Production Methods

30. Gill, R., Surface Finish and Roundness Error in Centreless Grinding. (Middlesex Polytechnic, UK)
31. Strode, I., and Goodfellow, S., The Effect of Electrodischarge Machining on the Surface Integrity and Mechamical Properties of an Alloy Steel. (Polytechnic of Wales, UK)
32. Weck, M., and Modemann, K., Surface Quality as a Function of the Static and Dynamic Machine Tool Behaviour in Process. (Aachen, WEST GERMANY)

### Engineering Function

33. Davis, E. J., Sullivan, P. J., and Stout, K. J., The Application of 3D Topography to Engine Bore Surfaces. (Birmingham, UK)
34. Dickson, G. R., Mcllhagger, R., and Miller, P. P., The Effeocts of Mould Surface Finish and Processing Conditions on the Surface Characteristics of Polymeric Injection Mouldings. (Ulster University, UK)
35. Griffiths, B. J., Manufacturing Surface Design and Monitoring for Performance. (Brunel University, UK)
36. McCool, J. I., Predicting Flash Temperature at Microcontacts. (SKF, USA)
37. Rao, N. A. B., and Raja, J., A Knowledge Based System for Selection of Surface Texture Parameters—A Preliminary Investigation. (Michigan Technological University, USA)
38. Tabenkin, A. N., and Parsons, F. G., Surface Analysis for Measurement of Pigment Agglomeration in Rubber. (Federal Products, USA)

## Figures and Tables

**Figure 1:**
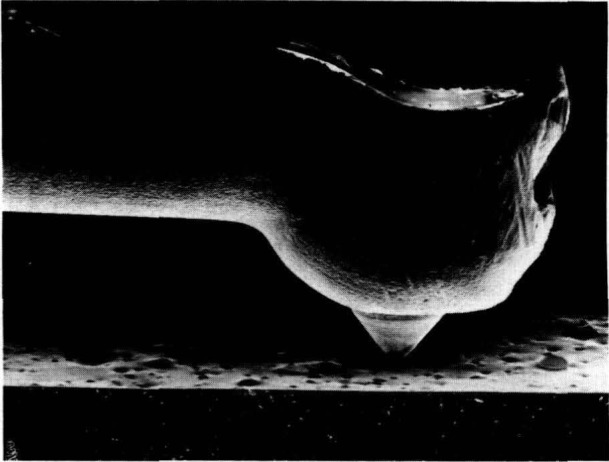
The presence of an agglomerate in a rubber sample is indicated by a peak or valley recorded on a chart as the stylus travels over the sample’s surface. SEM micrograph from Tabenkin and Parsons (paper 38).

**Figure 2:**
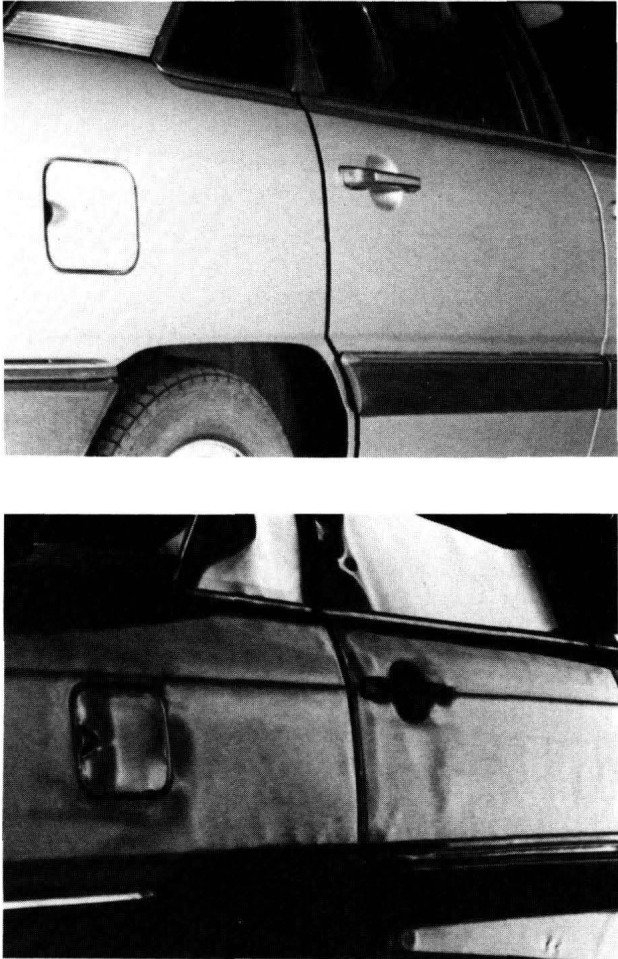
Comparison of a new car body viewed without D-sight (above) and with D-sight. The shadows in the images are caused by minute form deviations of the part. Taken from the work of Pryor et al. (paper 14).

**Figure 3:**
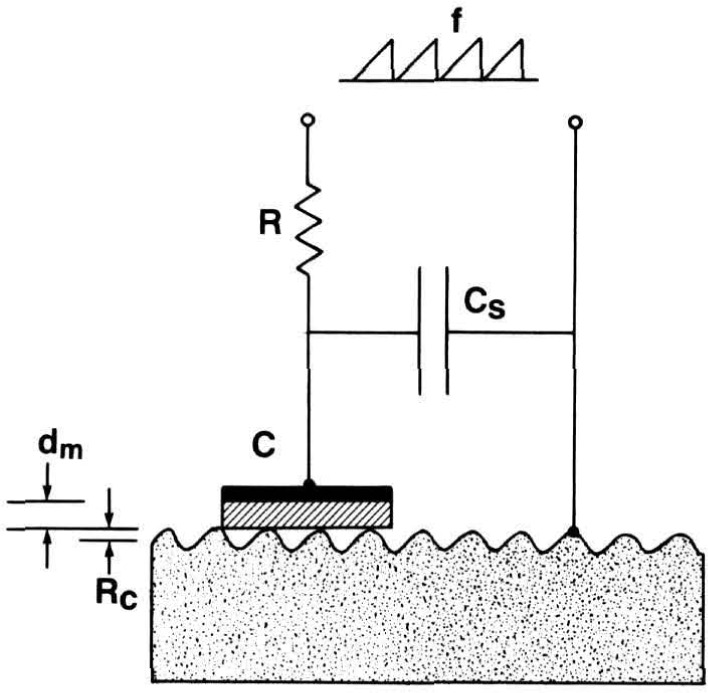
Schematic diagram of a capacitance instrument probing the topography of a rough surface. Capacitor C consists of the metallization (blackened) and the dielectric film (shaded) resting on the rough metal surface (speckled) beneath it. Taken from Lieberman et al. (paper 11).

**Figure 4:**
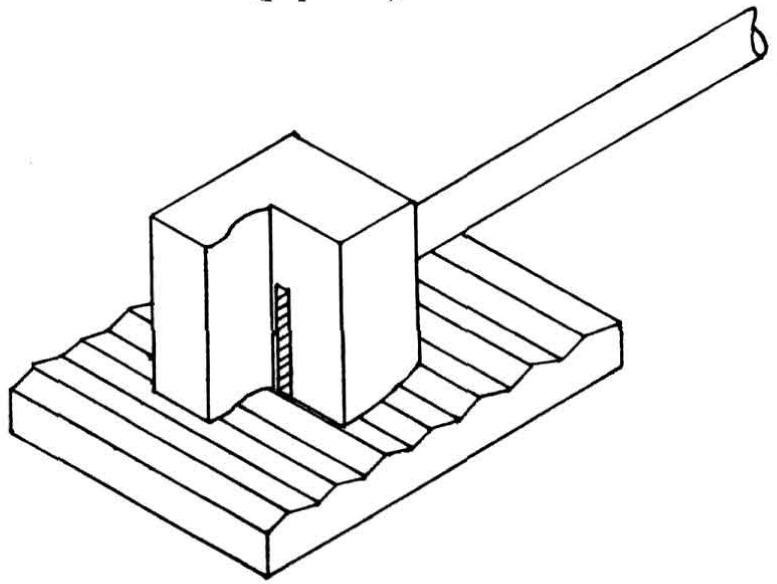
Cut-away view of the fringe field sensor. Taken from Garbini et al. (paper 7).

**Figure 5:**
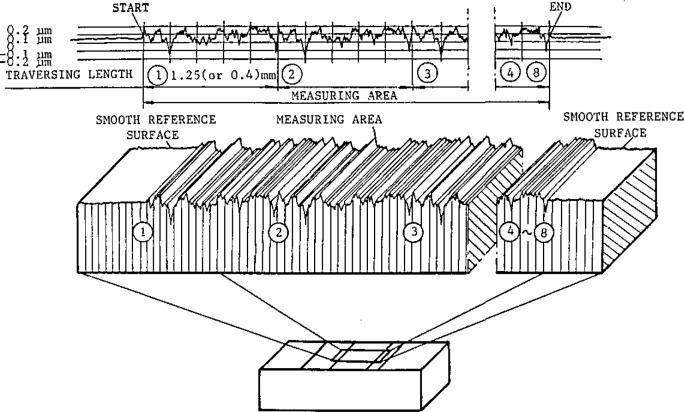
Detail of a roughness calibration specimen developed by Song (paper 21). It shows how the random roughness profile is repeated over the surface, thus achieving high uniformity.

**Figure 6:**
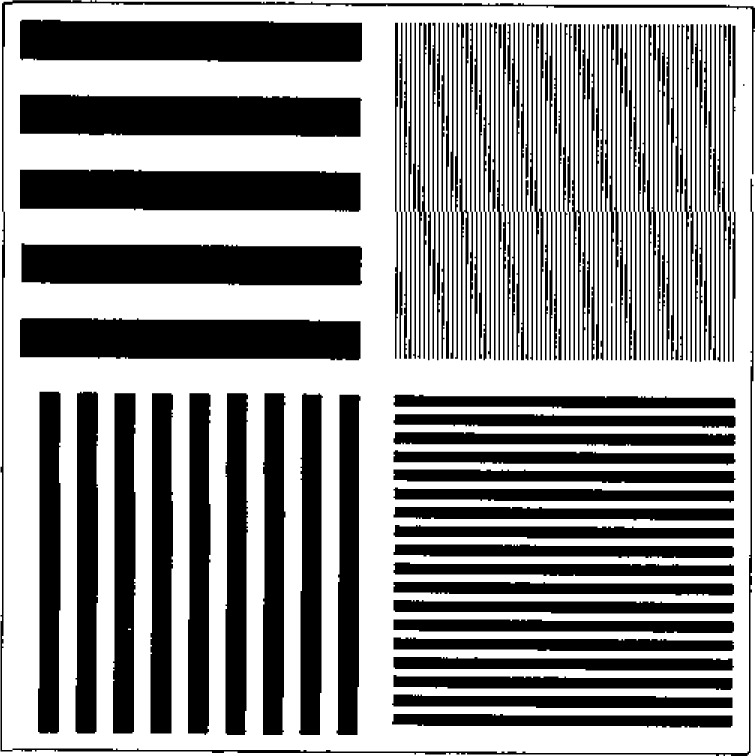
Layout of the “square wave” roughness specimen fabricated in silicon by Berger (paper 20). The specimen contains four patches having well-controlled pitches between the lines.

**Table 1:** Classification of engineering surface functions, important surface parameters, and notations on documents. Taken from J. Bielle (private communication) and presented by Rao et al. (paper 37)

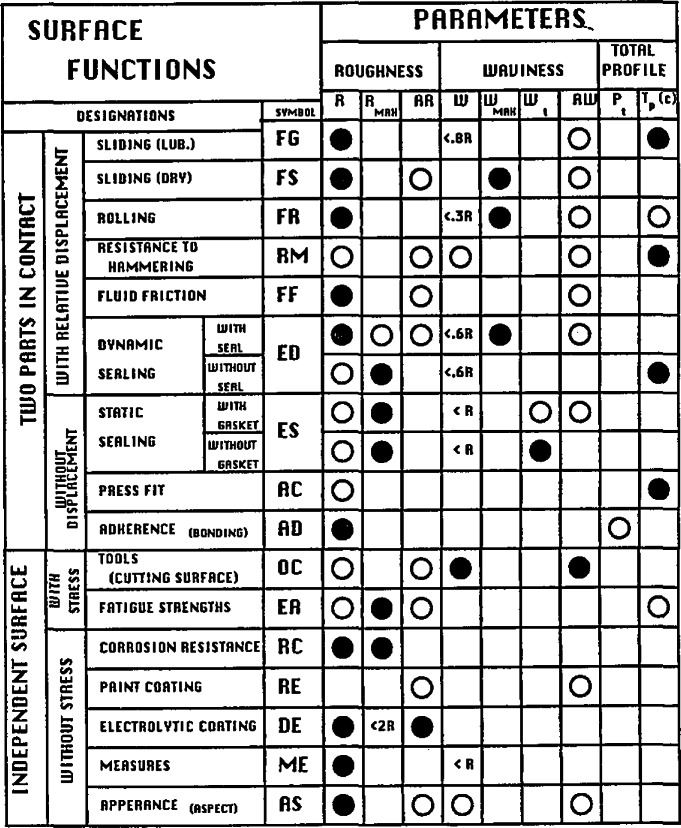

●: MOST IMPORTANT PARAMETERS TO BE SPECIFIED

○: SECONDARY PARAMETERS TO BE SPECIFIED DEPENDING ON FUNCTIONS

